# Open Joint1Presented at the Fourteenth Annual Meeting of the Iowa State Veterinary Medical Association, Des Moines, Iowa, February 11 and 12, 1902.

**Published:** 1902-04

**Authors:** J. Thomsen

**Affiliations:** Armstrong, Iowa


### OPEN JOINT.[1]

By J. Thomsen, V.S.,
ARMSTRONG, IOWA.

About December 1, 1901, a well-bred Percheron mare had, by kicking over a barbed-wire fence and becoming fast, received a wound in the anterior part of the tarsal joint. Considerable force had been used by the animal in attempting to free itself until it was finally assisted by its owner. The wound was in a horizontal direction, not over one and one-half inches in length, and seemingly not very deep. As there was no perceptible hemorrhage, and the mare walked as well as ever when led to the barn, the matter was thought to be of minor importance.

Upon a neighbor's advice a quantity of dry or air-slaked lime was pressed well into the wound daily. The mare was led a short distance to water twice a day, and showed no discomfort whatever for the first four days following the injury, but on the morning of the fifth day there was a change. She appeared much distressed, showed partial loss of appetite, and scarcely any weight could be carried on the affected limb. The owner, living at some distance from here, watched this condition for three days, and then asked for my assistance. As soon as seen I considered the case quite hopeless. This opinion was due somewhat to previous experience, having seen a number of similar cases. I always had felt justified in advising their destruction, which, as a rule, would be carried into effect.

There was a profuse flow of synovia from the opening, slightly to the inside of the median line, but which, on careful examination, appeared to extend backward and inward, possibly under the lower border of the tendinous slip of the flexor metatarsi which attaches to the cuneiform bone. A portion of the anterior border of the large cuneiform was felt to be devoid of covering and very rough. The animal was in the standing posture. The affected limb carried no

[1] Presented at the Fourteenth Annual Meeting of the Iowa State Veterinary Medical Association, Des Moines, Iowa, February 11 and 12, 1902.

weight whatever, but the most of the time was held a few inches off the floor, and constantly moved backward and forward in a dangling manner. She took but very little food, presented an anxious countenance, accelerated breathing, and a tucked-up abdomen. The circulation was considerably disturbed, with some elevation of temperature. The owner would not listen to my advice, but insisted on some sort of treatment. A dose of morphine sulphate was given, and later several doses of antifebrin were given at regular intervals. As to the wound treatment, I know of nothing effectual that I have ever employed, but having been impressed favorably with reports on the uses of the salt of silver in similar conditions, I concluded to use it here. Silver nitrate in solution of 1 to 125 was injected into the opening liberally. The exterior of the wound, which had at this time bulged out and become three or four times as wide as it was originally, was well sterilized and dusted over with an antiseptic powder, over which was placed absorbent cotton and a bandage—the latter applied loosely, yet well enough to keep the cotton in place. Slings were tried, but being objected to by the animal, a sort of frame was built around it, having two upright posts, two and one-half feet apart, immediately behind the animal. A cross-piece well wound with cloth was placed between the same and about three feet or more from the floor. The patient very soon discovered the comfort obtainable from this, for on the second day it rested for hours with its haunches pressed against the beam, finding great support in the contrivance. The wound was dressed regularly every six hours as above described, thus requiring attention during the night. The synovial discharge became perceptibly less on the third and fourth days, and on the seventh day had entirely ceased. The symptoms disappeared gradually and the appetite became excellent. An abscess now formed on the inner aspect of the joint. This was lanced and yielded very quickly to the same treatment. The at first greatly tumefied joint had now become much reduced, except around the lower region, where the swelling remained very hot and quite hard. The limb could not at this time carry much weight. The wound healed completely, leaving but a trifling scar. After considerable bathing for several days a blister was applied over both sides of the joint. This seemed to afford considerable relief, as the animal could now commence to use its leg more, and at this date, February 4, 1902, after a second application of a counter-irritant, appears to be making a good recovery.

